# Structure–Activity Relationship and Stage-Dependent Inhibition of Adipogenesis by Curcuminoid Derivatives in 3T3-L1 Cells

**DOI:** 10.3390/nu18081285

**Published:** 2026-04-18

**Authors:** Suzuna Araki, Yumi Ueda, Hinako Ayabe, Rio Otsuka, Kengo Kohama, Kouta Maenishi, Changsun Choi, Sung-Kwon Moon, Toshiya Masuda, Miwako Deguchi, Shigeru Saeki, DongHo Kim

**Affiliations:** 1 Laboratory of Molecular Nutrition and Metabolic Regulation, Department of Nutrition, Graduate School of Human Life and Ecology, Osaka Metropolitan University, 2-1-132 Morinomiya, Joto-ku, Osaka 536-8525, Japan; suzuna.araki0629@gmail.com (S.A.); yumi.ueda32@gmail.com (Y.U.); hinako.ayabe0524@gmail.com (H.A.); rio.otsuka0115@gmail.com (R.O.); kohamakengo@gmail.com (K.K.); maenishi19@gmail.com (K.M.); tmasuda@omu.ac.jp (T.M.); deguchi@omu.ac.jp (M.D.); shigeru.saeki@omu.ac.jp (S.S.); 2 Department of Food and Nutrition, Chung-Ang University, Anseong 456-756, Republic of Korea; cchoi@cau.ac.kr (C.C.); sumoon66@cau.ac.kr (S.-K.M.)

**Keywords:** curcuminoids, adipogenesis, 3T3-L1 cells, structure–activity relationships, bioactive compounds, in vitro evaluation, PPARγ, C/EBPα, KLF family

## Abstract

**Background/Objectives:** To address the limitations of natural curcumin, this study focuses on the functional evaluation of structurally optimized derivatives. We aimed to elucidate structure–activity relationships (SAR) and the stage-specific molecular mechanisms of adipogenesis inhibition using an in vitro cellular assay. **Methods:** Four novel curcuminoids were synthesized and evaluated in 3T3-L1 preadipocytes against natural curcumin (Curcuminoid I). Efficacy and mechanisms were assessed via cell viability assays, quantitative Oil Red O staining, and time-dependent transcriptional profiling (qPCR/Western blotting) of the KLF family and master regulators. **Results:** SAR analysis identified Curcuminoid III (symmetric 3,5-dimethoxy-4-hydroxy) as the most potent and safe candidate, whereas Curcuminoid IV exhibited cytotoxicity. Time-course analysis revealed a distinct step-wise inhibition mechanism wherein Curcuminoid III significantly upregulated the differentiation repressor KLF2 at the immediate-early phase. This rapid modulation effectively prevented the subsequent induction of pro-adipogenic factors, including KLF9, KLF15, PPARγ, and C/EBPα, in the mid-stage (3–5 d). Consequently, the expression of the maturation marker aP2 was robustly suppressed by the late stage (5–7 d). **Conclusions:** The symmetric 3,5-dimethoxy-4-hydroxy substitution pattern appears to confer strong anti-adipogenic activity to Curcuminoid III. Early modulation of the KLF2–PPARγ axis at the onset of differentiation may initiate a cascading inhibitory effect throughout the adipogenic program. These findings highlight the potential of structurally optimized plant-derived bioactive compounds as regulators of metabolic cell fate.

## 1. Introduction

The global prevalence of obesity has increased dramatically over recent decades and has become a major public health concern worldwide. Obesity is closely associated with metabolic disorders such as type 2 diabetes, cardiovascular disease, and hypertension, which together contribute substantially to global morbidity and mortality [1,2,3]. Adipose tissue expansion during obesity occurs through two distinct mechanisms: hypertrophy, characterized by an increase in adipocyte size, and hyperplasia, which involves the formation of new adipocytes through adipogenesis [4]. Although hypertrophic adipocytes are frequently associated with inflammation, hypoxia, and metabolic dysfunction, excessive adipocyte hyperplasia also contributes to adipose tissue remodeling and metabolic imbalance. Recent studies have further highlighted maladaptive white adipose tissue remodeling and impaired adipogenesis as central mechanisms linking obesity to systemic metabolic dysfunction [5,6]. Therefore, understanding the molecular regulation of adipogenesis is essential for developing strategies to prevent obesity-related metabolic diseases.

Adipogenesis is a highly coordinated cellular program that converts fibroblast-like preadipocytes into mature lipid-laden adipocytes. In 3T3-L1 cells, which are widely used as an in vitro model of white adipocytes, differentiation into adipocytes is initiated by hormonal stimulation with insulin, dexamethasone (DEX), and 3-isobutyl-1-methylxanthine (IBMX) [7,8]. Following the induction, preadipocytes undergo mitotic clonal expansion and activate a cascade of transcription factors that regulate adipocyte differentiation. Early transcriptional regulators such as C/EBPβ and C/EBPδ initiate the differentiation process and subsequently induce the expression of the master adipogenic transcription factors PPARγ and C/EBPα [9,10]. These master regulators then drive the expression of adipocyte-specific genes, including fatty acid-binding protein 4 (aP2/FABP4), which promotes lipid accumulation and terminal adipocyte maturation [11].

In addition to the classical PPARγ–C/EBPα axis, recent studies have highlighted the critical role of upstream transcriptional regulators in controlling adipocyte differentiation. Among these regulators, the Krüppel-like factor (KLF) family has emerged as a key transcriptional network governing adipogenesis. Members of the KLF family act as both positive and negative regulators of adipocyte differentiation. For example, KLF2 functions as a potent inhibitor of adipogenesis by suppressing PPARγ expression, whereas KLF5 promotes adipocyte differentiation during the early proliferative phase [12,13]. Other KLF members, including KLF9 and KLF15, are involved in the activation of adipogenic gene expression during the intermediate and late stages of adipocyte differentiation [14,15]. These findings indicate that the KLF transcription factor network acts as an upstream regulatory switch that determines whether adipocyte differentiation proceeds.

Natural compounds derived from plants have attracted considerable interest as potential modulators of adipocyte differentiation and metabolic health. Curcumin, a polyphenolic compound isolated from the rhizome of *Curcuma longa*, is widely known for its anti-inflammatory, antioxidant, and metabolic regulatory activities [16,17]. Curcumin has been increasingly discussed as a multi-target metabolic regulator with reported effects on adipocyte differentiation, inflammation, lipid handling, and whole-body energy metabolism [18,19,20,21]. Several studies have reported that curcumin suppresses adipocyte differentiation in 3T3-L1 cells by modulating signaling pathways involved in adipogenesis, including inhibition of mitotic clonal expansion and activation of Wnt/β-catenin signaling [22,23,24,25]. Despite these promising biological activities, the practical application of curcumin is limited by its poor bioavailability, rapid metabolic degradation, and chemical instability [16,17].

One strategy to overcome these limitations involves modifying the chemical structure of curcumin to generate more stable and biologically active derivatives. Curcuminoid derivatives with different substitution patterns on the aromatic rings provide an opportunity to examine how structural variations influence biological activity. Previous studies have demonstrated that the position and number of hydroxy and methoxy substituents strongly influence the antioxidant and biological activity of curcuminoid analogs [26,27,28]. In particular, the regio-specific placement of hydroxy and methoxy groups on the phenyl rings can affect radical stabilization and chemical reactivity, thereby influencing the biological activity of curcuminoid derivatives [26]. However, the structural determinants that distinguish potent anti-adipogenic activity from cytotoxic effects remain incompletely understood.

In the present study, we investigated the structure–activity relationships of curcuminoid derivatives using the 3T3-L1 adipocyte differentiation model. Four curcuminoid derivatives were synthesized and evaluated in comparison with natural curcumin. Based on preliminary screening, we hypothesized that Curcuminoid III, characterized by a symmetric 3,5-dimethoxy-4-hydroxy substitution pattern, would exhibit strong anti-adipogenic activity while maintaining low cytotoxicity. Furthermore, we examined whether this compound suppresses adipocyte differentiation through stage-specific regulation of the KLF transcriptional network and the downstream adipogenic cascade involving PPARγ, C/EBPα, and aP2.

## 2. Materials and Methods

### 2.1. Preparation of Bioactive Curcuminoid Derivatives

**Figure 1:**
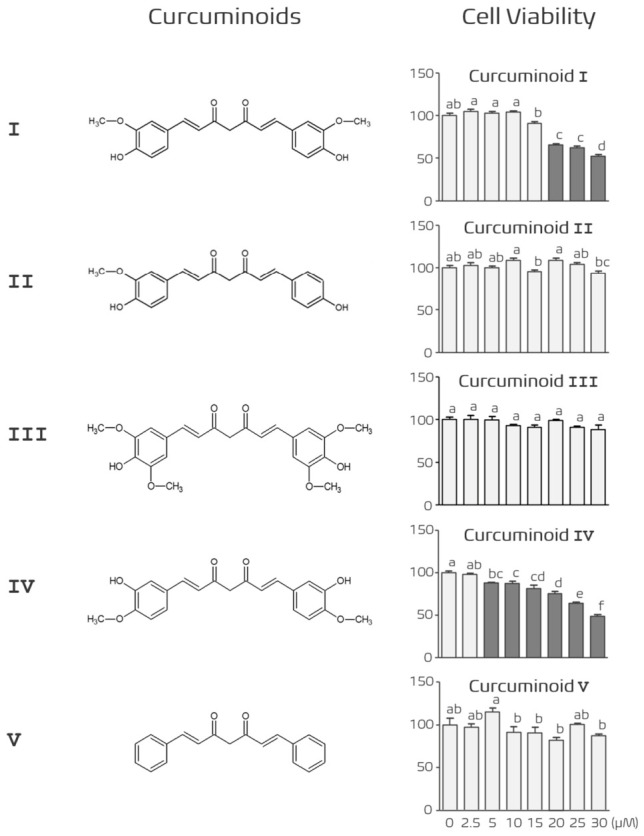
Chemical structures of curcuminoid derivatives and their effects on cell viability. (**Left**) Chemical structures of Curcuminoids I–V used in this study. Curcuminoid III contains a symmetric 3,5-dimethoxy-4-hydroxy substitution pattern. (**Right**) 3T3-L1 cells were treated with curcuminoids (0–30 μM) for 24 h, and cell viability was assessed using the MTT assay. Curcuminoids II, III, and V showed no significant cytotoxicity, whereas Curcuminoid I reduced viability at higher concentrations and Curcuminoid IV markedly decreased viability above 5 μM. Data are presented as mean ± SEM (*n* = 5 independent experiments). Different letters indicate statistically significant differences among groups (one-way ANOVA followed by Tukey’s post hoc test, *p* < 0.05).

To investigate the structure–activity relationships of plant-derived curcuminoids, a series of structurally modified curcuminoid analogs were prepared and evaluated in comparison with natural curcumin. Curcumin (natural curcumin) was purchased from FUJIFILM Wako Pure Chemical Corporation (Osaka, Japan) and consists of a mixture of curcuminoids, including curcumin, demethoxycurcumin, and bisdemethoxycurcumin. Curcumin (Curcuminoid I), chemically defined as 1,7-bis(4-hydroxy-3-methoxyphenyl)hepta-1,6-diene-3,5-dione, was used as the reference compound. The chemical structures of the curcuminoid analogs are shown in Figure 1 (left) and Supplementary Table S1.

These structural variations were designed to evaluate the relationship between substitution patterns on the aromatic rings and biological activity, particularly anti-adipogenic effects and cytotoxicity. In addition to this natural scaffold, four curcuminoid derivatives (Curcuminoids II–V) were chemically synthesized by modifying the substitution patterns of hydroxy and methoxy groups on the aromatic rings of the curcumin backbone in order to alter their physicochemical properties and biological activity. Curcuminoid derivatives (II–V) were synthesized as described previously [29,30]. The synthesized derivatives included Curcuminoid II (demethoxycurcumin), (1E,6E)-1-(4-hydroxy-3-methoxyphenyl)-7-(4-hydroxyphenyl) hepta-1,6-diene-3,5-dione; Curcuminoid III (5,5′-dimethoxycurcumin), (1E,6E)-1,7-bis(4-hydroxy-3,5-dimethoxyphenyl) hepta-1,6-diene-3,5-dione; Curcuminoid IV (isocurcumin), (1E,6E)-1,7-bis(3-hydroxy-4-methoxyphenyl) hepta-1,6-diene-3,5-dione; and Curcuminoid V, an unsubstituted parent scaffold derivative defined as (1E,6E)-1,7-diphenylhepta-1,6-diene-3,5-dione.

The structural identity of each compound was confirmed by spectroscopic analysis. Previous studies have demonstrated that the number and positional arrangement of hydroxy and methoxy substituents on the phenyl rings strongly influence the antioxidant capacity and biological activity of curcuminoid compounds [26,27,28,31]. Among the derivatives examined in this study, Curcuminoid III was designed with a symmetric 3,5-dimethoxy-4-hydroxy substitution pattern, which is predicted to enhance radical stabilization and redox activity. In contrast, Curcuminoid IV possesses a 3-hydroxy-4-methoxy (isovanillin-type) substitution pattern, which was included in the present analysis to evaluate structural features potentially associated with cytotoxicity.

### 2.2. In Vitro Model of Adipogenesis (Cell Culture and Differentiation)

Murine 3T3-L1 preadipocytes were obtained from the American Type Culture Collection (ATCC) and used as a standard in vitro model for adipocyte differentiation [7,8]. Cells were maintained in Dulbecco’s Modified Eagle’s Medium (DMEM) supplemented with 10% fetal bovine serum (FBS) and antibiotics at 37 °C in a humidified atmosphere containing 5% CO_2_. Adipocyte differentiation was induced according to a widely used protocol. Two days after reaching confluence, which was defined as Day 0 of differentiation, the cells were treated with a differentiation cocktail consisting of IBMX, DEX, and insulin in DMEM containing 10% FBS. After 48 h, designated as Day 2, the medium was replaced with DMEM containing insulin alone. From Day 4 onward, cells were maintained in DMEM supplemented with 10% FBS, and the culture medium was replaced every two days until Day 7 of differentiation. Curcuminoids were dissolved in dimethyl sulfoxide (DMSO) and added to the culture medium at concentrations ranging from 0 to 30 μM. Control cells were treated with the vehicle alone.

### 2.3. Cytotoxicity Screening (Cell Viability Assay)

To distinguish specific anti-adipogenic effects from nonspecific cytotoxicity, cell viability was evaluated using the MTT assay according to previously established methods [32]. Briefly, 3T3-L1 cells were cultured in DMEM supplemented with 10% FBS and treated with curcuminoids (I–V) at concentrations ranging from 0 to 30 μM for 24 h. Following incubation, MTT reagent was added to the culture medium to allow the formation of formazan crystals by metabolically active cells. The resulting crystals were dissolved, and the absorbance was measured at 570 nm using a microplate reader. Cell viability was expressed relative to untreated control cells. The final concentration of DMSO in all treatments was maintained at 0.05% (*v*/*v*), and an equivalent concentration of DMSO was used as a vehicle control.

### 2.4. Functional Evaluation of Lipid Accumulation (Oil Red O Staining)

The effect of curcuminoids on adipocyte differentiation was evaluated by Oil Red O staining at Day 7 after induction of differentiation, which is a widely used method for assessing lipid accumulation in adipocytes [33,34]. At the end of the differentiation period, cells were fixed with 10% formalin and stained with Oil Red O solution to visualize intracellular lipid droplets. Microscopic images were obtained to assess lipid accumulation qualitatively. For quantitative analysis, the retained dye was extracted with isopropanol, and the absorbance was measured at 490 nm using a spectrophotometer. Lipid accumulation was expressed relative to untreated differentiated control cells.

### 2.5. Stage-Specific Transcriptional Profiling (qPCR)

To investigate the molecular mechanisms underlying the anti-adipogenic effects of curcuminoids, stage-specific gene expression analysis was performed using quantitative real-time polymerase chain reaction (qPCR). Total RNA was extracted from cells harvested at the indicated time points using Sepasol-RNA I Super G (Nacalai Tesque, Kyoto, Japan; 09379-97), followed by phase separation with chloroform (FUJIFILM Wako Pure Chemical Corporation, Osaka, Japan; 038-02606). RNA precipitation was carried out using isopropanol (FUJIFILM Wako Pure Chemical Corporation; 166-04836) in the presence of Ethachinmate (Nippon Gene, Tokyo, Japan; 312-01791), and the RNA pellet was washed with 75% (*v*/*v*) ethanol (FUJIFILM Wako Pure Chemical Corporation; 057-00456 diluted with distilled water). The purified RNA was dissolved in TE buffer (10 mM Tris-HCl, 1 mM EDTA; Nippon Gene; 314-90021).

To eliminate genomic DNA contamination, RNA samples were treated with DNase I (Invitrogen, Carlsbad, CA, USA; 18047-019) in the presence of 10× DNase I Reaction Buffer, and the reaction was terminated using EDTA solution (Invitrogen; 18068-015). Time points for harvesting included early stages (0, 0.5, 1, 2, 4, 6, 12, 24, and 48 h after induction), an intermediate stage (Day 3), and a late stage corresponding to mature adipocytes (Day 7). These time points were selected to capture the sequential transcriptional events associated with adipocyte differentiation [9,10,35,36]. Complementary DNA (cDNA) was synthesized from 1 μg of total RNA using a reverse transcription system containing 20 mM dNTP mix (TaKaRa Bio Inc., Shiga, Japan; 4026–4029), 80 μM random primers (TaKaRa; 3801), 0.1 M DTT, and 5× First-Strand Buffer (Invitrogen; 18080-044). Reverse transcription reactions were performed using a GeneAmp^®^ PCR System 9700 (Applied Biosystems, Foster City, CA, USA).

Quantitative PCR was performed using PowerUp™ SYBR^®^ Green Master Mix (Applied Biosystems; A25743) on an ABI StepOnePlus Real-Time PCR System (Applied Biosystems). The genes analyzed included early regulatory transcription factors such as KLF2, KLF3, KLF4, KLF5, KLF7, C/EBPβ, and C/EBPδ, as well as intermediate regulators including KLF9, KLF15, PPARγ, and C/EBPα. In addition, the expression of aP2, the adipocyte maturation marker, was evaluated during the late stage of differentiation. Gene expression levels were normalized to 36B4 as an internal control and calculated using the 2^−ΔΔCt^ method. The primer sequences used in this study are listed in Supplementary Table S2. Each experiment was performed using three independent biological replicates, and each sample was analyzed in technical triplicate.

### 2.6. Protein Expression Analysis (Western Blotting)

To validate transcriptional changes at the protein level, Western blot analysis was performed using cells harvested at key stages of adipocyte differentiation. Cells were lysed in RIPA buffer supplemented with a protease inhibitor cocktail (Nacalai Tesque, Kyoto, Japan), and protein concentrations were determined using a BCA Protein Assay Kit (Thermo Fisher Scientific, Waltham, MA, USA).

Equal amounts of protein were separated by SDS-PAGE and transferred onto PVDF membranes. The membranes were blocked with 5% skim milk in Tris-buffered saline containing 0.1% Tween-20 (TBST) for 1 h at room temperature and then incubated overnight at 4 °C with the following primary antibodies: C/EBPβ (Santa Cruz Biotechnology, Dallas, TX, USA; sc-7962; 1:200), C/EBPδ (sc-515028; 1:100), PPARγ (sc-7273; 1:200), C/EBPα (sc-365318; 1:100), KLF15 (sc-271675; 1:100), and β-actin (sc-47778; 1:200). After washing with TBST, membranes were incubated with HRP-conjugated goat anti-mouse IgG secondary antibody (Santa Cruz Biotechnology; sc-2005; 1:5000) for 1 h at room temperature. Immunoreactive bands were detected using ECL™ Prime Western Blotting Detection Reagents (GE Healthcare, Chicago, IL, USA; RPN2232) and visualized using a ChemiDoc Imaging System (Bio-Rad, Hercules, CA, USA). Band intensities were quantified using Image Lab™ Software (Bio-Rad, version 6.1), and target protein levels were normalized to β-actin. All Western blot experiments were performed using three independent biological replicates.

### 2.7. Statistical Analysis

All quantitative data are presented as mean ± SEM. Experiments were conducted with three independent biological replicates, and qPCR analyses were performed in technical triplicate for each biological sample. Statistical analyses were conducted using GraphPad Prism software (version 9.0; GraphPad Software, San Diego, CA, USA). For comparisons between two groups, Student’s unpaired *t*-test was used. For experiments involving multiple treatment groups, including cell viability assays, Oil Red O staining analysis, and gene expression time-course experiments, statistical significance was determined using one-way analysis of variance (ANOVA) followed by Tukey’s post hoc test for multiple comparisons. Differences were considered statistically significant at *p* < 0.05.

## 3. Results

### 3.1. Cytotoxicity Evaluation of Curcuminoid Derivatives in 3T3-L1 Cells

Before examining the anti-adipogenic effects of curcuminoid derivatives, we first evaluated their potential cytotoxicity in 3T3-L1 cells. Cells were cultured in DMEM containing 10% FBS and treated with increasing concentrations (0–30 μM) of five curcuminoid derivatives (Curcuminoids I–V). Cell viability was assessed using the MTT assay.

Curcuminoids II, III, and V did not significantly affect cell viability within the tested concentration range, indicating that these compounds were not cytotoxic up to 30 μM. In contrast, Curcuminoid I reduced cell viability at concentrations above 20 μM, whereas Curcuminoid IV markedly decreased cell viability even at concentrations above 5 μM (Figure 1). These results indicate that certain curcuminoid derivatives exhibit cytotoxic effects depending on their chemical structure. Based on these findings, Curcuminoids II, III, and V were selected for further evaluation of their anti-adipogenic activity.

### 3.2. Screening of Curcuminoid Derivatives for Inhibition of Adipocyte Differentiation

To investigate the anti-adipogenic potential of curcuminoid derivatives, 3T3-L1 preadipocytes were induced to differentiate in the presence of Curcuminoids I–V. Lipid accumulation in differentiated adipocytes was evaluated by Oil Red O staining, a widely used method for quantifying adipocyte differentiation.

**Figure 2:**
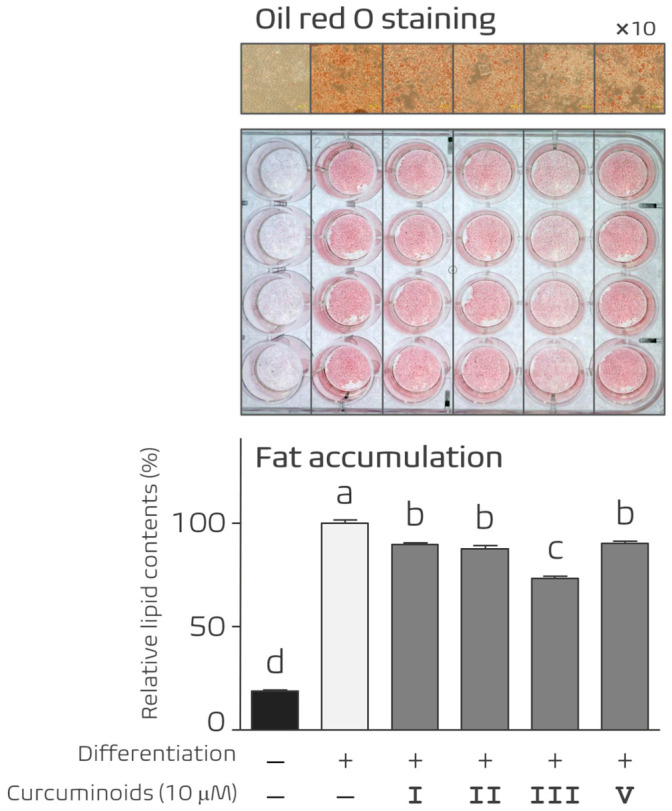
Effects of curcuminoid derivatives on adipocyte differentiation. 3T3-L1 preadipocytes were induced to differentiate in the presence or absence of Curcuminoids I, II, III, and V. Lipid accumulation was evaluated by Oil Red O staining at Day 7. Representative images are shown. Curcuminoids I, II, III, and V reduced lipid accumulation, with Curcuminoid III showing the strongest inhibitory effect without detectable cytotoxicity. Data are presented as mean ± SEM (*n* = 5 independent experiments). Different letters indicate statistically significant differences among groups (one-way ANOVA followed by Tukey’s post hoc test, *p* < 0.05).

Treatment with several curcuminoid derivatives reduced lipid accumulation compared with untreated differentiated cells (Figure 2). Curcuminoids I, II, III, and V inhibited adipocyte differentiation to varying degrees. Among the derivatives tested, Curcuminoid III exhibited the strongest inhibitory effect on lipid accumulation while maintaining low cytotoxicity. These findings identified Curcuminoid III as the most promising compound for further mechanistic analysis.

### 3.3. Dose-Dependent Inhibition of Adipocyte Differentiation by Curcuminoid III

To further characterize the anti-adipogenic effect of Curcuminoid III, we examined whether its inhibitory activity was dose-dependent. 3T3-L1 cells were induced to differentiate and treated with increasing concentrations of Curcuminoid III during the differentiation period.

**Figure 3:**
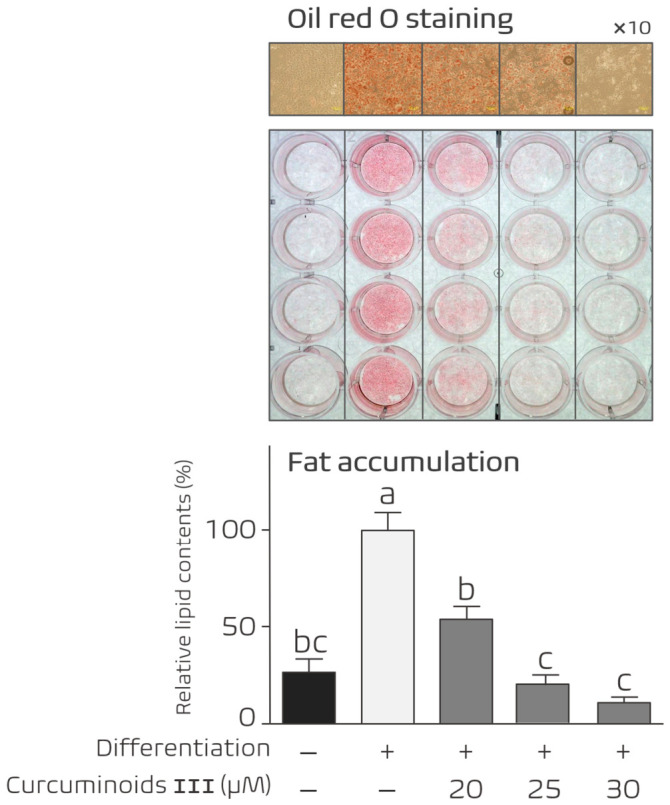
Dose-dependent inhibition of adipocyte differentiation by Curcuminoid III. 3T3-L1 cells were induced to differentiate in the presence of increasing concentrations of Curcuminoid III. Lipid accumulation was assessed by Oil Red O staining at Day 7. Curcuminoid III reduced lipid accumulation in a dose-dependent manner, and 25 μM was selected for subsequent experiments. Data are presented as mean ± SEM (*n* = 5 independent experiments). Different letters indicate statistically significant differences among groups (one-way ANOVA followed by Tukey’s post hoc test, *p* < 0.05).

Oil Red O staining revealed that lipid accumulation decreased progressively as the concentration of Curcuminoid III increased (Figure 3). Quantitative analysis of extracted Oil Red O dye confirmed a significant reduction in lipid accumulation in a concentration-dependent manner. A concentration of 25 μM produced a strong inhibitory effect without affecting cell viability. Based on these results, 25 μM Curcuminoid III was selected as the standard experimental concentration for subsequent analyses.

### 3.4. Curcuminoid III Regulates Transcriptional Factors During the Early Stage of Adipocyte Differentiation

To investigate the molecular mechanisms underlying the anti-adipogenic activity of Curcuminoid III, we analyzed the expression of transcription factors during the early stage of adipocyte differentiation. Total RNA and protein were collected at multiple time points after induction of differentiation.

**Figure 4:**
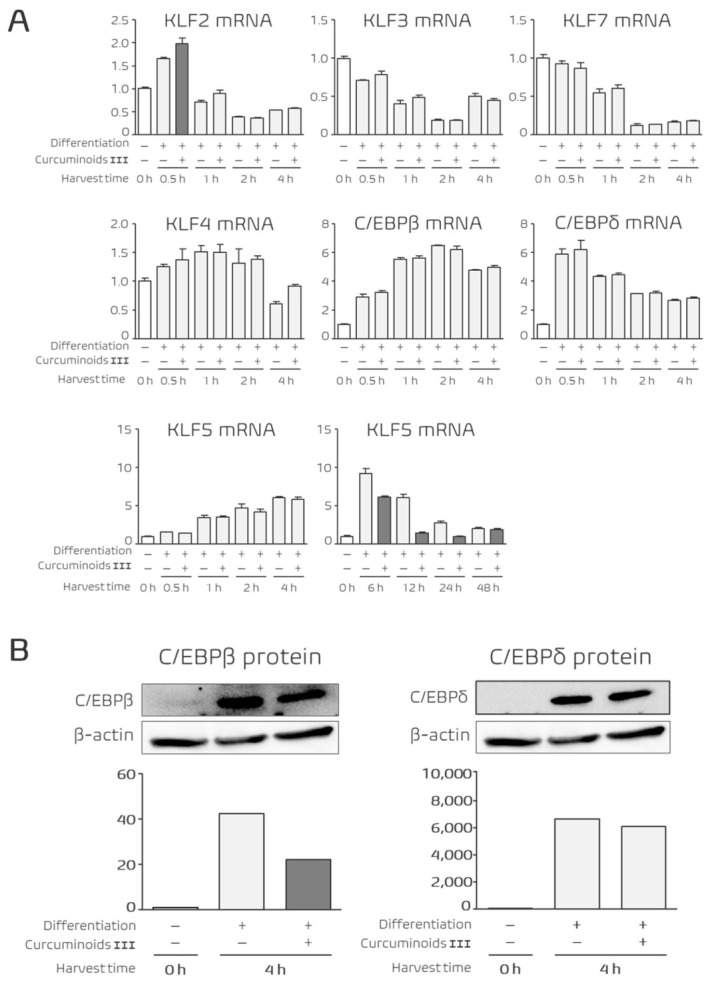
Effects of Curcuminoid III on early-stage transcriptional regulators during adipocyte differentiation. 3T3-L1 cells were treated with or without 25 μM Curcuminoid III and harvested at indicated time points (0–48 h). (**A**) mRNA expression levels of KLF family members were analyzed by quantitative real-time PCR. Curcuminoid III increased KLF2 expression at 0.5 h and suppressed KLF5 expression at 6 h. Data are presented as mean ± SEM (*n* = 4 independent experiments). (**B**) Protein expression levels of C/EBPβ and C/EBPδ were analyzed by Western blotting. Curcuminoid III reduced C/EBPβ expression at 4 h, whereas C/EBPδ remained unchanged. Western blot experiments were performed using three independent biological replicates, and β-actin and target proteins were detected on the same membrane.

Quantitative real-time PCR analysis revealed that Curcuminoid III significantly increased KLF2 mRNA expression at 0.5 h after induction of differentiation (Figure 4A). KLF2 has been reported to function as an inhibitory regulator of adipocyte differentiation [12,35]. In contrast, the expressions of KLF3 and KLF7 did not change significantly during this early stage. In addition, Curcuminoid III significantly suppressed KLF5 mRNA expression at 6 h after induction (Figure 4A).

Western blot analysis further confirmed that the protein level of C/EBPβ, a master adipogenic regulator, was markedly reduced at 4 h, whereas C/EBPδ remained unchanged following Curcuminoid III treatment (Figure 4B). These results indicate that Curcuminoid III alters early transcriptional events associated with the initiation of adipocyte differentiation.

### 3.5. Curcuminoid III Suppresses Adipogenic Regulators During the Mid Stage of Differentiation

We next examined whether Curcuminoid III affects transcriptional regulators during the intermediate stage of adipocyte differentiation. Gene expression was analyzed at Day 3 after induction, which corresponds to the mid stage of adipogenesis.

**Figure 5:**
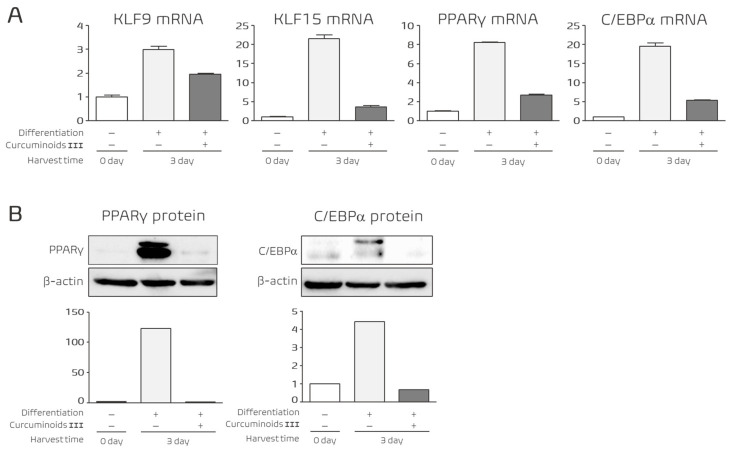
Effects of Curcuminoid III on adipogenic regulators during the mid stage of differentiation. 3T3-L1 cells were treated with 25 μM Curcuminoid III and harvested at Day 3. (**A**) mRNA expression levels of KLF9, KLF15, PPARγ, and C/EBPα were analyzed by quantitative real-time PCR. Curcuminoid III significantly reduced the expression of these genes. Data are presented as mean ± SEM (*n* = 4 independent experiments). (**B**) Protein expression levels of PPARγ and C/EBPα were analyzed by Western blotting, confirming suppression of adipogenic regulators. Western blot experiments were performed using three independent biological replicates.

At this stage, Curcuminoid III significantly reduced the mRNA expression of KLF9, KLF15, PPARγ, and C/EBPα (Figure 5A). These transcription factors play critical roles in promoting adipocyte differentiation and activating the adipogenic transcriptional cascade [10,14,15].

Western blot analysis further confirmed that the protein levels of the master adipogenic regulators PPARγ and C/EBPα were markedly reduced following Curcuminoid III treatment (Figure 5B). These results indicate that Curcuminoid III suppresses the activation of the central adipogenic transcriptional program during the intermediate stage of differentiation.

### 3.6. Curcuminoid III Inhibits Adipocyte Maturation During the Late Stage of Differentiation

We next investigated whether the inhibitory effects of Curcuminoid III persist during the late stage of adipocyte differentiation. Gene expression analysis was therefore performed at Day 7 after induction, corresponding to the stage of adipocyte maturation.

**Figure 6:**
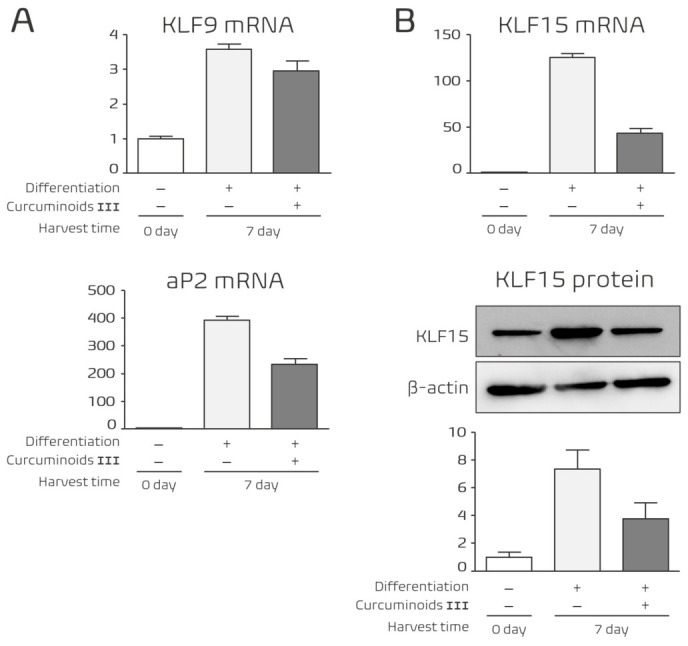
Effects of Curcuminoid III on adipocyte maturation during the late stage of differentiation. 3T3-L1 cells were treated with 25 μM Curcuminoid III and harvested at Day 7. (**A**) mRNA expression levels of KLF9 and aP2 were analyzed by quantitative real-time PCR. Curcuminoid III significantly reduced the expression of these genes. Data are presented as mean ± SEM (*n* = 4 independent experiments). (**B**) mRNA and protein expression levels of KLF15 were analyzed by quantitative PCR and Western blotting, respectively, indicating suppression of adipocyte maturation. Western blot experiments were performed using three independent biological replicates.

During this late stage, Curcuminoid III continued to suppress adipogenic gene expression. The mRNA levels of KLF9, KLF15, and the adipocyte maturation marker aP2 were significantly decreased compared with control cells (Figure 6A,B). Because aP2 encodes a fatty acid-binding protein associated with lipid accumulation during adipocyte maturation [36], its suppression indicates that Curcuminoid III inhibits the terminal differentiation and maturation of adipocytes.

In addition, Western blot analysis demonstrated that KLF15 protein expression was reduced in Curcuminoid III-treated cells (Figure 6B). These findings indicate that Curcuminoid III suppresses adipocyte maturation and lipid storage during the late stage of differentiation.

### 3.7. Structural Considerations of Curcuminoid Derivatives in Relation to Anti-Adipogenic Activity

Finally, we compared the biological activities of the curcuminoid derivatives to explore potential structure–activity relationships. Although several derivatives inhibited adipocyte differentiation, Curcuminoid III exhibited the strongest anti-adipogenic activity without detectable cytotoxicity. In contrast, Curcuminoid IV showed significant cytotoxicity at relatively low concentrations (Figure 1). Structural comparison suggests that differences in substitution patterns on the phenyl rings may influence the biological activity of these molecules.

**Figure 7:**
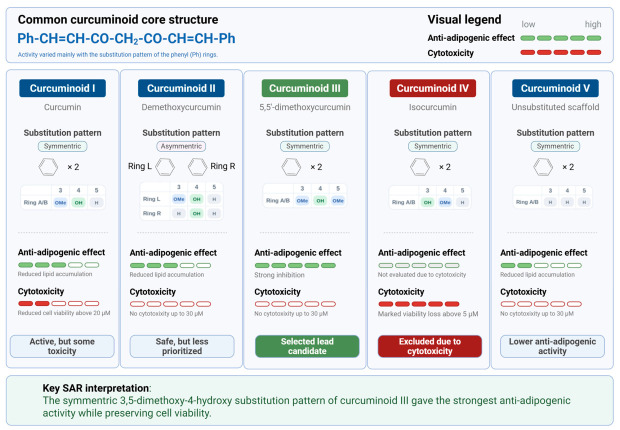
Structure–activity relationship of curcuminoid derivatives based on methoxy/hydroxy substitution patterns and their effects on adipocyte differentiation. Curcuminoid derivatives were compared according to the methoxy and hydroxy substitution patterns on the phenyl rings. Among the compounds tested, Curcuminoid III, characterized by a symmetric 3,5-dimethoxy-4-hydroxy substitution pattern, exhibited the strongest inhibitory effect on adipocyte differentiation with minimal cytotoxicity. In contrast, Curcuminoid IV, possessing a 3-hydroxy-4-methoxy (isovanillin-type) substitution pattern, showed increased cytotoxicity and was excluded from further functional evaluation. Other derivatives displayed moderate inhibitory effects with low cytotoxicity. These findings indicate that specific methoxy/hydroxy substitution patterns are associated with differential anti-adipogenic activity and cellular toxicity. The schematic also summarizes the proposed mechanism in which Curcuminoid III induces early KLF2 expression and suppresses downstream adipogenic regulators, including PPARγ, thereby inhibiting adipocyte differentiation.

Curcuminoid IV contains both hydroxyl and methoxy substituents on the aromatic ring, which may alter the physicochemical properties of the compound. In contrast, Curcuminoid III retains strong anti-adipogenic activity while maintaining cell viability (Figure 2 and Figure 3). Previous studies have shown that the regio-specific placement of hydroxy and methoxy groups in curcuminoids can influence antioxidant and biological activities [26,27]. A schematic summary of the structures and relative biological activities of the curcuminoid derivatives is presented in Figure 7.

## 4. Discussion

Curcuminoids are plant-derived polyphenolic compounds widely recognized for their antioxidant, anti-inflammatory, and metabolic regulatory activities. In the present study, we investigated the anti-adipogenic effects of structurally modified curcuminoid derivatives using the 3T3-L1 adipocyte differentiation model. Among the derivatives examined, Curcuminoid III exhibited the strongest inhibitory effect on adipocyte differentiation while maintaining low cytotoxicity. Time-course analysis revealed that this compound suppresses adipogenesis in a stage-dependent manner by modulating transcriptional regulators associated with adipocyte differentiation. In particular, Curcuminoid III induced the inhibitory transcription factor KLF2 during the early stage of differentiation and subsequently suppressed downstream adipogenic regulators, including KLF9, KLF15, PPARγ, and C/EBPα, ultimately leading to reduced expression of the maturation marker aP2.

One notable finding of this study is the rapid induction of KLF2 during the early phase of adipocyte differentiation. KLF2 has been identified as a negative regulator of adipogenesis that suppresses PPARγ expression and prevents adipocyte maturation [12,35]. In contrast, KLF5 functions as a positive regulator of adipocyte differentiation during the early proliferative phase by activating downstream transcriptional programs [13]. The simultaneous induction of KLF2 and suppression of KLF5 observed in the present study suggests that Curcuminoid III interferes with an early regulatory step that determines whether preadipocytes proceed toward adipogenic commitment. In addition, the reduction in C/EBPβ protein levels further supports the idea that Curcuminoid III disrupts the initiation phase of the adipogenic transcriptional cascade [9,36]. Together, these findings indicate that Curcuminoid III suppresses adipogenesis by altering early transcriptional events that regulate the initiation of the differentiation program.

In addition to its effects on early transcriptional regulators, Curcuminoid III also suppressed adipogenic gene expression during the intermediate and late stages of differentiation. The expression of PPARγ and C/EBPα, which function as master regulators of adipocyte differentiation, was markedly reduced following treatment with Curcuminoid III. These transcription factors drive the expression of adipocyte-specific genes involved in lipid accumulation and metabolic function [1,11]. Consistent with this transcriptional cascade, the expression of the adipocyte maturation marker aP2 was also significantly decreased. These results suggest that Curcuminoid III does not merely delay adipocyte differentiation but rather suppresses the transcriptional cascade required for adipocyte maturation and lipid storage.

Previous studies have reported that curcumin inhibits adipocyte differentiation through multiple mechanisms, including suppression of mitotic clonal expansion and modulation of Wnt/β-catenin signaling pathways [22,23,25]. Activation of Wnt signaling maintains preadipocytes in an undifferentiated state by suppressing adipogenic transcription factors such as PPARγ and C/EBPα [37]. The present findings extend these observations by demonstrating that structurally modified curcuminoid derivatives may regulate adipocyte differentiation through early modulation of the KLF transcriptional network [38,39]. This upstream regulatory layer may represent an additional mechanism through which curcuminoid derivatives influence adipogenic cell fate decisions.

Similar anti-adipogenic effects have been reported for several food-derived bioactive compounds in the 3T3-L1 adipocyte differentiation model. For example, gymnemic acids isolated from *Gymnema inodorum* have been shown to suppress lipid accumulation and reduce the expression of adipogenic regulators such as PPARγ and C/EBPα [40]. Lotus (*Nelumbo nucifera*) leaf–derived preparations have likewise been reported to inhibit adipocyte differentiation through modulation of metabolic signaling pathways [41]. In addition, plant-derived phenolic compounds including extracts of *Euscaphis japonica* and isoeugenol have been shown to inhibit adipogenesis at relatively early stages of differentiation [42,43]. Proanthocyanidins and related phenolic compounds have also been reported to exert anti-lipogenic effects in 3T3-L1 preadipocytes, further supporting the role of plant-derived molecules in regulating adipocyte biology [44]. Moreover, recent comprehensive analyses of turmeric-derived phenolics have highlighted their capacity to modulate adipogenesis and metabolic signaling across multiple systems [45]. These studies collectively suggest that diverse food-derived molecules converge on the adipogenic transcriptional cascade. In contrast to these studies, which primarily reported suppression of the canonical adipogenic regulators PPARγ and C/EBPα, the present findings suggest that Curcuminoid III acts at an earlier regulatory level by inducing the inhibitory transcription factor KLF2 and suppressing KLF5. This observation indicates that curcuminoid derivatives may influence adipocyte differentiation by modulating transcriptional events upstream of the classical adipogenic cascade.

Another important aspect of this study is the relationship between chemical structure and biological activity among curcuminoid derivatives. Unlike natural curcumin, which exists as a mixture of curcuminoids in turmeric, the derivatives examined in this study (Curcuminoids II–V) are structurally defined compounds synthesized to systematically evaluate structure–activity relationships [26,27,28,46,47]. Demethoxycurcumin (Curcuminoid II) and bisdemethoxycurcumin are known naturally occurring constituents of turmeric, typically present as minor components relative to curcumin, whereas the symmetric 3,5-dimethoxy-4-hydroxy–substituted Curcuminoid III and related analogs are not known to occur in natural sources and should be considered synthetic derivatives. Therefore, although some curcuminoid structures retain dietary relevance, the present study primarily focuses on chemically optimized analogs designed to enhance biological activity and stability beyond naturally occurring compounds.

Although several derivatives inhibited adipocyte differentiation, Curcuminoid III exhibited the most favorable balance between anti-adipogenic activity and low cytotoxicity. Structural comparison suggests that the symmetric 3,5-dimethoxy-4-hydroxy substitution pattern may contribute to enhanced biological activity. Previous studies have shown that the antioxidant and biological activities of curcuminoids are strongly influenced by the position and number of hydroxy and methoxy substituents on the aromatic rings [27,28]. Recent studies on plant-derived phenolics have likewise demonstrated that relatively small structural differences can substantially alter anti-lipogenic or anti-adipogenic activity, supporting the view that substitution patterns are critical determinants of biological potency [40,41]. These structural features may influence the stabilization of phenolic radicals and the redox properties of curcuminoid molecules. The present findings therefore support the concept that subtle structural modifications can significantly influence the biological activity of curcuminoid derivatives.

In addition, Curcuminoid IV exhibited relatively strong cytotoxicity compared with other derivatives. This observation may be associated with its structural features, particularly the 3-hydroxy-4-methoxyphenyl (isovanillin-type) substitution pattern. Similar structural motifs are found in highly cytotoxic compounds such as combretastatin A4, suggesting that specific substitution patterns may contribute to enhanced cytotoxic effects. Further studies will be required to clarify the molecular mechanisms underlying this activity. Conversely, the symmetric 3,5-dimethoxy-4-hydroxyphenyl (syringyl-type) structure present in Curcuminoid III may contribute to its favorable balance between strong anti-adipogenic activity and low cytotoxicity. Similar syringyl-type phenolic structures have been reported in plant-derived compounds with relatively low toxicity and potent biological activity, supporting the importance of this substitution pattern.

Although reduced lipid accumulation was observed in the present study, we cannot completely exclude the potential contribution of changes in cell proliferation. However, no significant reduction in cell viability was observed under the experimental conditions used for adipogenesis assays, suggesting that the observed inhibitory effects are not primarily due to a reduction in cell number or proliferative capacity. The concentrations used in this study were selected based on cell viability assays, further supporting the notion that the anti-adipogenic effects of Curcuminoid III are not attributable to nonspecific cytotoxicity. Nevertheless, additional analyses using proliferation markers will be required to clearly distinguish between anti-adipogenic and anti-proliferative effects.

In the 3T3-L1 differentiation model, lipid accumulation is generally associated with adipocyte hypertrophy. Nevertheless, the possible contribution of hyperplasia cannot be excluded. Further investigation will be required to determine whether the observed effects are primarily mediated through changes in lipid droplet size, adipocyte number, or both. Quantitative analysis of lipid droplet size would provide additional mechanistic insight, although this was not assessed in the present study. Thus, the present findings are more likely to reflect suppression of adipocyte differentiation rather than changes in adipocyte number.

Beyond the specific molecular mechanisms described above, the present findings also provide a broader perspective on how early transcriptional regulators influence adipogenic cell fate decisions. Although adipocyte differentiation is often described as a sequential transcriptional program, systems biology studies have suggested that cell fate transitions can be governed by regulatory checkpoints that operate in a threshold-like manner. Transcriptional networks controlling cell differentiation can generate bistable regulatory states that determine whether cells commit to differentiation programs [48]. In this context, the rapid induction of KLF2 and suppression of pro-adipogenic regulators observed in this study may reflect modulation of an early regulatory step within the adipogenic transcriptional network.

Interestingly, our previous study identified a high-molecular-weight mucosal protein, IgGFcγBP, as a lactoferrin-binding partner in the intestine, suggesting the presence of an interface through which dietary-derived factors interact with biological regulatory systems [49]. Lactoferrin has been increasingly recognized as a bioactive dietary factor involved in adiposity and metabolic regulation, further supporting the concept that food-derived molecules can influence systemic metabolic homeostasis [50,51]. Although the present study focuses on adipocyte differentiation, the ability of structurally defined bioactive compounds to modulate early regulatory events may represent a shared principle across different physiological contexts. Furthermore, early-stage regulatory alterations have been observed in metabolic systems prior to overt phenotypic changes [52,53], supporting the idea that early transcriptional responses may play a decisive role in shaping downstream cellular outcomes. In line with previous reports demonstrating that curcumin suppresses adipogenesis through canonical pathways [22], our findings extend this concept by highlighting an immediate-early transcriptional regulatory layer.

Finally, while the present study demonstrates the anti-adipogenic potential of curcuminoid derivatives in vitro, their stability, metabolism, and bioavailability in vivo remain unclear. In particular, the metabolic fate of Curcuminoid III and its potential active metabolites should be investigated in future studies to evaluate its physiological relevance.

The metabolic stability and potential biotransformation of Curcuminoid III remain to be elucidated. Given its structural similarity to curcumin, Curcuminoid III may undergo reductive metabolism and phase II conjugation (e.g., glucuronidation and sulfation) in vivo, as reported for curcumin [16], although this requires experimental validation.

Several limitations of this study should also be acknowledged. First, the experiments were conducted using a single in vitro adipocyte differentiation model. Although the 3T3-L1 system is widely used to study adipogenesis, further studies using additional cellular models or in vivo systems will be necessary to confirm the physiological relevance of these findings. Second, although the present study suggests a relationship between curcuminoid structure and biological activity, the number of derivatives examined was limited. Future studies involving a larger library of curcuminoid analogs may provide a more comprehensive understanding of structure–activity relationships. Finally, identification of the direct molecular targets of curcuminoid derivatives will be important for clarifying the precise mechanisms through which these compounds regulate adipocyte differentiation.

## 5. Conclusions

In summary, the present study demonstrates that structurally modified curcuminoid derivatives suppress adipocyte differentiation through stage-dependent modulation of adipogenic transcriptional regulators. Among the compounds examined, Curcuminoid III exhibited the strongest inhibitory activity while maintaining low cytotoxicity. Mechanistically, this compound induced the inhibitory transcription factor KLF2 and suppressed key adipogenic regulators including KLF9, KLF15, PPARγ, and C/EBPα, thereby disrupting the transcriptional cascade required for adipocyte maturation.

These findings highlight the importance of early transcriptional regulatory events in determining adipogenic cell fate and suggest that structurally optimized curcuminoids can function as potent modulators of adipocyte differentiation. In a broader context, our results support the concept that dietary bioactive molecules, including polyphenols and other functional food components, may influence metabolic cell fate decisions by modulating regulatory checkpoints within transcriptional and signaling networks. Understanding how such nutritional signals influence these regulatory systems may contribute to the development of functional food strategies for the prevention of obesity-related metabolic disorders.

## Data Availability

The data presented in this study are available on request from the corresponding author due to institutional restrictions and ongoing research.

## Supplementary Materials

The following supporting information can be downloaded at: https://www.mdpi.com/article/10.3390/nu18081285/s1. Supplementary Table S1: Curcuminoid derivatives (Curcuminoids I–V) used in this study. Supplementary Table S2: Primer sequences used for quantitative real-time PCR analysis of adipogenic transcription factors during 3T3-L1 differentiation.

## Abbreviations

The following abbreviations are used in this manuscript:

KLF	Krüppel-like factor
PPARγ	Peroxisome proliferator-activated receptor gamma
C/EBP	CCAAT/enhancer-binding protein
aP2/FABP4	Fatty acid-binding protein 4
DMEM	Dulbecco’s Modified Eagle’s Medium
FBS	Fetal bovine serum
IBMX	3-isobutyl-1-methylxanthine
DEX	Dexamethasone
SAR	Structure–activity relationship
