# Supplementary material for: Structure–Activity Relationship and Stage-Dependent Inhibition of Adipogenesis by Curcuminoid Derivatives in 3T3-L1 Cells
Source: Nutrients. 2026 Apr 18;18(8):1285. doi: 10.3390/nu18081285 (PMC13118917; doi:10.3390/nu18081285)
Supplement: Supplementary file 1 [file nutrients-18-01285-s001.zip › Sup Table S2_curcuminoids_Araki et al.pdf]

Supplementary Table S2. Primer sequences used for quantitative real-time PCR analysis.

Gene	*NCBI Accession (mRNA)*	*Sequence*		*Expected length (bp)*
36b4	NM_007475	Forward	5’-CACTGGTCTAGGACCCGAGAAG-3’	73
		Reverse	5’-GGTGCCTCTGGAGATTTTCG-3’	
Klf2	NM_008452	Forward	5’- TTTTGGATCTTTGGAGGAATCAC -3’	51
		Reverse	5’- CTACCCTATCCCCAGCCACA -3’	
Klf3	NM_008453	Forward	5’- TCATACAGGAGAAAAGCCGTACAA -3’	51
		Reverse	5’- TTCCACGTGCAGCCTTCC -3’	
Klf4	NM_010637	Forward	5’- GACCAGATGCAGTCACAAGTCC -3’	51
		Reverse	5’- TGGCATGAGCTCTTGATAATGG -3’	
Klf5	NM_009769	Forward	5’- TGGCGATTCACAACCCAAA -3’	51
		Reverse	5’- TTGGCGAATTAACTGGCAGAG -3’	
Klf6	NM_011803	Forward	5’- AGCTTTTGGCCGTGAGCA -3’	52
		Reverse	5’- CAACCATCCCACCCACTAACA -3’	
Klf7	NM_033563	Forward	5’- ACAACTTGTCCACGACACCG -3’	51
		Reverse	5’- TCCTCCAGGGATGGCAAAG -3’	
Klf9	NM_010638	Forward	5’- GGAAAATCCTCCCATCTTAAAGC -3’	56
		Reverse	5’- AAGGGCCGTTCACCTGTATG -3’	

Klf15	NM_023184	Forward	5’- GCAGCCATACCACATGTTGC -3’	51
		Reverse	5’- ACATCGCTGTCATCCTCCG -3’	
Pparγ	NM_011146	Forward	5’- CCACCAACTTCGGAATCAGC -3’	51
		Reverse	5’- AGTGGTCTTCCATCACGGAGA -3’	
C/ebpα	NM_007678	Forward	5’- GGGCAAAGCCAAGAAGTCG -3’	51
		Reverse	5’- CGTACCCGGTACTCGTTGCT -3’	
C/ebpβ	NM_009883	Forward	5’- TCGGGACTTGATGCAATCC -3’	64
		Reverse	5’- GTTGCGTCAGTCCCGTGTC -3’	
C/ebpδ	NM_007679	Forward	5’- AACACGGGAAAGCATGACTAATTC-3’	51
		Reverse	5’- GTCAGCCTACTCTGGGATCACAC -3’	
aP2	NM_024406	Forward	5’- TGGGAACCTGGAAGCTTGTCTC -3’	116
		Reverse	5’- GCTGATGATCATGTTGGGCTTG -3’	
