# Supplementary material for: Structure–Activity Relationship and Stage-Dependent Inhibition of Adipogenesis by Curcuminoid Derivatives in 3T3-L1 Cells
Source: Nutrients. 2026 Apr 18;18(8):1285. doi: 10.3390/nu18081285 (PMC13118917; doi:10.3390/nu18081285)
Supplement: Supplementary file 1 [file nutrients-18-01285-s001.zip › Sup Table S1_curcuminoids_Araki et al.pdf]

Supplementary Table S1. Structural characteristics of curcuminoid derivatives (Curcuminoids I–V) used in this study.

Compound	*Common Name*	*Chemical Description*	*Substitution Pattern (Phenyl Rings)*	*Structural Feature*	*Expected Biological Property*
Curcuminoid I	Curcumin	1,7-bis(4-hydroxy-3-methoxyphenyl) hepta-1,6-diene-3,5-dione	4-OH, 3-OCH_3 (both rings)	Natural reference structure	Baseline activity; limited by low bioavailability
Curcuminoid II	Demethoxycurcumin	(1E,6E)-1-(4-hydroxy-3-methoxyphenyl)-7-(4-hydroxyphenyl) hepta-1,6-diene-3,5-dione	One ring: 4-OH, 3-OCH_3 (other ring: 4-OH only)	Asymmetric substitution	Moderate biological activity
Curcuminoid III	5,5′-Dimethoxycurcumin	(1E,6E)-1,7-bis(4-hydroxy-3,5-dimethoxyphenyl) hepta-1,6-diene-3,5-dione	4-OH, 3,5-$(OCH_3)_2$ (both rings)	Symmetric, methoxy-rich structure	Strong anti-adipogenic activity with low cytotoxicity
Curcuminoid IV	Isocurcumin	(1E,6E)-1,7-bis(3-hydroxy-4-methoxyphenyl) hepta-1,6-diene-3,5-dione	3-OH, 4-OCH_3 (both rings)	Isovanillin-type substitution	Increased cytotoxicity
Curcuminoid V	Unsubstituted analog	(1E,6E)-1,7-diphenylhepta-1,6-diene-3,5-dione	No OH or OCH_3 (groups)	Parent scaffold (no substituents)	Reduced biological activity
